# Health in crises. Migration, austerity and inequalities in Greece and Europe: introduction to the supplement

**DOI:** 10.1093/eurpub/cky223

**Published:** 2018-11-21

**Authors:** Terje A Eikemo, Lydia Avrami, Jennifer Cavounidis, Aliki Mouriki, Anna Gkiouleka, Courtney L McNamara, Theoni Stathopoulou

**Affiliations:** 1 Department of Sociology and Political Science, Centre for Global Health Inequalities Research (CHAIN), Norwegian University of Science and Technology (NTNU), Trondheim, Norway; 2 National Centre for Social Research, Athens, Greece; 3 Department of Economics, Athens University of Economics and Business, Athens, Greece; 4 Department of Sociology, University of York, York, UK

## Abstract

This introduction summarizes and discusses the main findings of the supplement *‘Health in crises. Migration, austerity and inequalities in Greece and Europe’* to the European Journal of Public Health. The supplement applies data from the ESS (2014) health module in combination with the MIGHEAL study, which is a new source of data on the Greek population specially designed to examine health inequalities among and between migrants and natives. This has enabled the authors of the nine articles that constitute this supplement to address several pressing issues about the distribution of health and its determinants in Greece and other European countries. The main finding of the present supplement is the exceptionally high rates of reported depressive symptoms across the whole population residing in Greece and particularly among women. Levels of unmet need for healthcare were also found to be alarmingly high in Greece compared with other European countries, suggesting that the crisis and subsequent austerity policies may have impacted the provision of healthcare services and access to healthcare for broad sections of the population, whether native or migrant.

## Introduction

The economic recession and persistent austerity measures have put welfare state provisions and universal health coverage under severe pressure in Greece.^1^ As a result, new groups at risk of poverty and social exclusion (i.e. low paid, unemployed, informal care providers) have emerged amongst both the native and the migrant population, while rates of unmet care needs are growing within the population.^2^ In this context, this supplement of the *European Journal of Public Health* entitled *‘Health in crises. Migration, austerity and inequalities in Greece and Europe’* presents results from the MIGHEAL study, which is a new source of data on the Greek population specially designed to examine health inequalities among and between migrants and natives.

The MIGHEAL study constitutes a major step forward not only for within-country comparisons among population sub-groups in Greece, but also for cross-national comparisons of health outcomes and their social, behavioural and health care-related determinants at the European level. These comparisons are made possible because the MIGHEAL study was developed in parallel with the European Social Survey (ESS) Round 7.^3^

The authors of the nine articles of the present supplement address several pressing issues regarding health status and its determinants in a European country, which has been severely impacted by a prolonged social and economic crisis. More specifically, the objectives of the MIGHEAL study were to (i) examine the self-rated health status of migrant and native populations and identify social inequalities in physical and mental health, as well as their determinants; (ii) investigate migrants’ health needs and healthcare usage compared to that of the non-migrant population and identify the relevant barriers to access and utilization of health services; (iii) provide evidence at the national level regarding social inequalities in health comparable with the pan-European documentation provided by the ESS and (iv) exploit this urgently needed information in policy recommendations to combat health inequalities and social exclusion more generally, thereby contributing to the integration of migrants in the Greek society.^4^

In Introduction section, we first discuss the Greek context in relation to migration and we elaborate on why the distinction between migrants and Greek-born in Greece is important for the examination of health status and its determinants. Next, we highlight the most important findings of the articles of this supplement followed by a discussion with some concluding remarks.

### The migration profile of Greece

Greece, traditionally a migrant-sending country, became a migrant-hosting country during the early 1990s, after the fall of socialist regimes in Central and Eastern Europe. Albania emerged as the most important source country, accounting for 58% of all foreigners in the 2001 census. During the next decade, between 2001 and 2011 the registered foreign population in the country increased by 20%. According to the 2011 Greek census,^5^ out of a total population of 10. 816. 286 there were 712 879 third-country nationals (non-EU citizens), accounting for 6.6% of the total population, and 199 121 European Union (EU) nationals who were not citizens of Greece (1.8% of the total population). Foreign nationals are mainly from Albania (480 851) and other Balkan countries, namely Bulgaria (75 917) and Romania (46 524) as well as from South Asian countries (Pakistan, Bangladesh, and Afghanistan). In the 1990s and 2000s, heavy migrant inflows corresponded to the intense economic activity of that period and the demand for flexible, cheap and often non-regulated employment.^6^

The global economic crisis that emerged in 2008 resulted in significant changes in the migratory profile of Greece. High unemployment rates that disproportionately affect migrants have forced many of them to move to other destinations while trapping others in a precarious situation. Acquiring a residence permit in Greece presupposes being employed and paying social insurance contributions. Hence, a long period of unemployment for a migrant is translated into loss of entitlement to residence permit and consequently, to healthcare and welfare services.

Also since 2008, increasing numbers of migrants and refugees from Asian and African regions have been arriving in the country. Even before the abrupt increase in refugee arrivals in 2015, Greece was one of the main EU entry points for refugees and third-country migrants, due to its geographical position and porous borders with Turkey; namely the Evros River at the north part of the country and the extensive island coastline in the East Aegean Sea. During 2015, the phenomenon reached a climax as approximately 857 000 migrants and refugees entered Greece through its maritime border in urgent need of humanitarian and medical assistance.^7^

As a result of inadequate legal migration channels to Europe, of European policies which attempt to deter unauthorized migration^8^ and of the inability of the Greek state to process asylum claims effectively,^6^ the majority of third-country migrants have been unable to secure their status in the country. A valid stay permit, the acquisition of which involves a long and tedious process, is required in order to qualify as a ‘documented’ migrant. The greatest number of valid stay permits (∼600 000) was reported in December 2010. Since then, a decrease in the number of valid stay permits has been observed, although a slight increase was noted from 2015 until the end of September 2016, when the number of valid stay permits reached 572 574.^9^

At the policy level, Greek migration and integration policies since the early 1990s can be generally characterized as reactive rather than proactive, focussing on redressing previous failures, a primary example being the three programmes for ‘regularization of undocumented’ migrants that were carried out in the late 1990s and mid-2000s.^10^ In 2014, legislation known as the Immigration and Social Integration Code (law 4251/2014) was approved in an attempt to codify all previous legislation and regulate the entry, stay, and social integration of other country nationals in Greece. An important feature of the Code is that it allows residence permit renewals for other country nationals who have lost their authorized status, or for those who, due to unemployment spells, were previously ineligible to renew their work permits.^11^ Despite its progressive aspects, the Code does not seem to take account of the heterogeneity of the migrant population in Greece, while its implementation is more often impeded by state bureaucracies.

With respect to the employment of migrants and their integration in the labour market, it should first be noted that before the crisis, unemployment rates among migrants were lower than among natives, contrary to the experience of most EU countries. Subsequent to the crisis, however, migrant unemployment rates increased much more than those of natives, primarily due to their concentration in manual jobs in sectors of the economy that were severely affected by the crisis, such as construction in the case of men and private households in the case of women.^7^ According to OECD data, unemployment rates for foreign-born men and women in 2015 were 31.4 and 32.8%, respectively, while the corresponding rates for native-born men and women were 20.9 and 28.7%.^12^

Finally, considering migrants’ healthcare access, it seems again that there is no coherent and effective policy framework. Since the 1990s, access to public health care services and medicines has been to a greater or lesser extent entangled with residence permits and social insurance and tax contributions.^13^ In 2000, a circular issued by the Ministry of Health and Welfare stated that non-European migrants have the same rights to health care access with European citizens as long as they have an active stay/work permit that implies also the possession of social insurance. For individuals who have received refugee status or another official form of international protection, the same entitlements apply without the condition of a valid work permit.^14^ This regulation however leaves multiple groups of migrants without healthcare coverage (except for emergency care) including asylum seekers with a pending asylum claim, migrants who cannot renew their residence permit due to long-term unemployment, and third country migrants who have been unable to acquire an ‘authorized’ status. Beyond the legal regulations, what happens in everyday practice is even less encouraging. The frequent changes in the country’s central administration, the continuous shifts in policy, the overwhelming budget cuts in public health expenditure, the lack of culturally sensitive healthcare practices and increased xenophobia make access to health services a challenge and the navigation of the healthcare system almost impossible.^13^

### Health status of migrants

Relevant surveys have been conducted in Greece, mostly by healthcare professionals and to a lesser extent by social scientists. Documentation of healthcare provision, healthcare needs and self-reported conditions is scarce and inconclusive. Available surveys are usually small in scale and restricted either to a specific geographical area in Greece^15^ or to a specific topic, such as mental health disorders or the prevalence of specific diseases in migrant populations.^16^ Evidence of the association between economic hardship and the deterioration of the mental health status of the general population in Greece has been provided by nationwide cross-sectional surveys in 2008 and 2009^17^^,^^18^ as well as by the University Mental Health Research Institute in 2011, focussing primarily on depression and suicidality during the recession.^19^ Similar findings at a European level were reported by Stuckler et al.^20^

Evidence suggests that second-generation migrants face problems rather frequently due to their origin when accessing health services in public hospitals. More specifically, a significant number felt discriminated against by nursing staff (21.5%), doctors (16.6%) or administrative staff (16.6%) during hospitalization or outpatient visits.^21^ Men and women report similar experiences of discrimination among second-generation immigrants in Greece. Furthermore, second-generation migrants with Albanian citizenship are less discriminated compared with those with citizenship corresponding to former socialist countries of Central and Eastern Europe.^22^

A meta-analytical overview of data on migrant integration policies in the areas of health, welfare and social security has also shown that migrants are at high risk of social exclusion and unmet health needs due to their poor residence conditions. The crisis of welfare provision during the recession years in Greece has further increased this risk.^22^ The lack of reliable information among the migrant population on both the availability of health services and the terms of accessibility was shown to be a critical factor determining healthcare usage by migrants.^23^

These considerations clearly demonstrate the multiple ways that migrants in Greece are kept in a disadvantaged social position and highlight the need of a new health survey which accounts for the imbalance between migrants and non-migrants that is able to document potential relationships of health inequality between and within those groups. As the articles included in this supplement demonstrate, the MIGHEAL study responds to this need and presents the most comprehensive and recent snapshot of the Greek health situation both from a domestic and pan-European perspective.

The nine articles share a common approach to inequalities in self-rated health and non-communicable diseases (NCDs), where the aim of the analyses is both to describe within-country (between migrants and natives) and cross-national inequalities in the socially structured experience of different determinants and outcomes. Our approach is informed by Whitehead’s work on health inequalities, which understands the unequal experience of health as being avoidable, unfair and unjust.^24^ Moreover, we take an intersectional approach, in which various dimensions of social positioning (such as migration status, education, ethnic origin or gender) are seen in combination. The articles also share an analytical approach, which ensures comparability between them. More specifically, we apply the same dataset (the MIGHEAL survey, sometimes in combination with the ESS), the same measures of key variables and the same estimation techniques.

## Highlights

In this section, we make a summary of all articles and highlight some of the most important results of the supplement. The second article by Cavounidis^25^ contextualizes the MIGHEAL study in the wake of the economic crisis and discusses the rationale for defining migrant and non-migrant groups in this study. In the third article, Stathopoulou et al.^26^ present the MIGHEAL survey together with prevalences of several key health determinants and health outcomes in comparison with other 21 European countries. The results of this article not only provide the most comprehensive descriptive overview of health determinants and outcomes in Greece to date, they also reveal some additional and rather alarming findings. For example, the authors report exceptionally high levels of depressive symptoms in Greece, and particularly among women. Further, they observe comparatively high levels of unmet needs, risk behaviours (such as smoking and binge drinking), economic hardship and provision of unpaid care both among men and women. The fourth article by Yfantopoulos and Chantzaras^27^ examines the magnitude of income-related health inequalities among both Greek-born and migrant populations in Greece, finding substantial inequalities favouring the better-off among both population sub-groups, particularly regarding mental health problems. The fifth article by Stathopoulou et al.^28^ examines self-reported depressive symptoms in further detail. Their analysis does not confirm previous evidence that migrants are at an increased risk for depressive symptoms in Greece. More specifically, while depressive symptoms in Greece among migrants are very high, migrants are not at an increased risk compared to the native population. Age, gender and perceived discrimination appear to be more decisive determinants of self-reported depressive symptoms than migrant background. The sixth study by Gkiouleka et al.^29^ broadens the scope further by exploring self-reported depressive symptoms in the light of migration status, gender, childhood experiences, socioeconomic factors and social support across European countries that have been unevenly affected by the economic crisis. They find that the crisis’ impact on self-reported depressive symptoms among migrants and non-migrants has not been uniform across socio-demographic contexts. Moreover, they suggest that the impact of migration status on depressive symptoms is not reduced to socio-economic marginalisation and perceived discrimination but rather it is closely entwined with them as well as with early life experiences and gender, thus urging us to adopt an intersectional research perspective. In this line, the seventh article by Eikemo et al.^30^ focuses on gender inequalities in NCDs in the light of migration status and ethnic origin reflecting an intersectional understanding of social positioning. Findings show that both among non-migrant and migrant groups women report substantially higher rates of NCDs and that the largest part of those gender inequalities are explained by occupational factors for both groups. However, the range of gender inequalities in NCDs is subject to both migration status and ethnic origin. The eighth paper by Rapp et al.^31^ assesses the relationship between social integration, in terms of social contact and social trust, and one’s individual self-reported health. The authors find that migrant status plays a moderating role in the relationship between social integration and health, yet, this finding holds true only for the cross-national European comparison based on the ESS 2014 survey data. The final paper by Kentikelenis^32^ discusses how the already residual Greek welfare state model became further undermined by the economic crisis, leading to a deterioration of health outcomes.

## Discussion

The key finding of this supplement is the alarming rate of self-reported depressive symptoms across all population groups in Greece. This finding is supported by and explored in multiple contributions of the supplement.^26–29^ For example, Stathopoulou et al.^28^ identified a mixture of risk factors associated with the high depression rates, including financial strain, provision of unpaid care, as well as experiences of domestic conflict or economic hardship in childhood. Such symptoms were less prevalent among migrants than non-migrants and were exceptionally high among Greek women. The observed association between childhood experiences and depressive symptoms is of particular importance, as it may suggest that negative childhood experiences of conflict in the family of origin or deprivation may be sparked off or reinforced by negative experiences in adult life. This connects to both the theory of the ‘accumulation’ of disadvantage and life-course approaches^33^ but also to psychological theories regarding the long lasting impact of early life experiences that render traumatised people more vulnerable than others.

This key finding may be related to the more frequently reported NCDs among migrant women and native women in Greece.^30^ However, the frequent reporting of depressive symptoms among women cannot be solely explained by the occurrence of NCDs, as the prevalence of NCDs is very low in Greece in comparison to other European countries. Rather, the comparatively higher levels of unmet health care needs in Greece, as well as of economic hardship, risk behaviours and provision of unpaid care (all of which could be crisis-related), confirm previous studies that have found a negative health impact of the crisis particularly among women. Women in the Greek society are particularly burdened by gender discrimination both outside and inside the labour market.^34^ As for the latter, women’s employment rates were among the lowest in EU27 before the economic crisis, while during the recession women’s job opportunities were limited to a greater extent than those of men.^35^ Contrary to men, the largest decline in employment rates for women are found among those highly educated.^36^ Women are over-represented in sectors where flexible working patterns, lower earnings and diminished pension rights prevail, as well as in unpaid work in family businesses.^36^ Although the dual-earning model has gained ground during the recession due to the austerity’s impact on male employment at the initial stages of the crisis, women still carry the burden of domestic and care work,^37^ a role that further hinders their working potential and independence from the family and adds extra physical and psychological demands. When it comes particularly to migrant women, concentration in flexible, low-skilled and low-paid sectors without social security is even higher. The income loss incurred by the prolonged austerity has resulted in a significant reduction in the demand for domestic services, previously covered mainly by migrant women and combined with the deregulation of employment conditions and the shrinking of welfare provisions have further deteriorated the socioeconomic status for a large share of migrant women.^36^ These developments highlight how women have been disproportionately affected by the economic recession and seem to play an important role in widening the gender gap in depression as has been argued elsewhere.^38^

Greece has been experiencing a severe economic and social crisis since 2008. In order to provide information vital to the design of policies to overcome the consequences of this crisis, it is of the utmost importance to study population health and health inequalities. The MIGHEAL study contributed to establishing a complex pattern of inequalities between population sub-groups. However, the potential of the MIGHEAL study goes far beyond within-country comparisons. The study provides the most recent and comprehensive overview of self-reported conditions and their determinants in Greece within a European context,^26^ which can and should be further elaborated in future comparative studies.

Although repeated cross-sectional studies in Greece would be needed to establish causality, our findings are in line with prior research that has associated the prolonged crisis in Greece with adverse health outcomes. When compared with levels recorded in the ESS, exceptionally high levels of self-reported depressive symptoms were found in Greece, particularly among women. Levels of unmet need for healthcare were also found to be alarmingly high in Greece compared with ESS countries, suggesting that the crisis and subsequent austerity policies may have impacted the provision of healthcare services and access to healthcare for broad sections of the population, whether native or migrant.

## Funding

This article is part of the project ‘Health Inequalities among Migrant Population (MIGHEAL)’, co-funded by the Financial Mechanism of the European Economic Area (EEA). Programme on Diversity, Inequalities and Social Inclusion, 2009–2014 (Grant number: GR-07: 3807). The Project was led by the National Centre for Social Research in Athens Greece, in collaboration with the Norwegian University for Science and Technology, in Trondheim Norway, and operated by the General Secretariat for Research and Technology, Ministry of Education, Research and Religious Affairs in Greece. This article is also part of the Norwegian Research Council funded project ‘ESS7 Health Module: Equality in Access to Health Care’ (project number 228990).

*Conflicts of interest*: None declared.
